# Dignity Conserving Therapy: An Intervention for Addressing Psychosocial and Existential Distress in Patients with Serious Illness

**DOI:** 10.1089/pmr.2022.0016

**Published:** 2022-09-16

**Authors:** Jessica Weng, Deirdre R. Pachman, Ellen Wild, Cory J. Ingram

**Affiliations:** ^1^ Mayo Clinic College of Medicine and Science, Mayo Clinic Alix School of Medicine, Rochester, Minnesota, USA.; ^2^ Department of General Internal Medicine and Mayo Clinic, Rochester, Minnesota, USA.; ^3^ Center for Palliative Medicine, Mayo Clinic, Rochester, Minnesota, USA.; ^4^ Department of Family Medicine, Mayo Clinic, Rochester, Minnesota, USA.

**Keywords:** dignity therapy, meaning-centered psychotherapy, oncology palliative care, palliative care

## Abstract

Patients with serious illnesses may experience existential and psychosocial distress contributing to their pain and suffering. Addressing existential distress is challenging and may require a multidisciplinary approach. Often, providers feel uncomfortable or ill equipped to care for patients suffering from this distress. In the sample case, the patient has a life-limiting disease and is concerned about his family forgetting him, experiencing loss of dignity and narrative foreclosure. Loss of dignity is sensing hopelessness and worthlessness and a loss of self-determination. Narrative foreclosure is the premature conviction that one's life story has effectively ended. Beneficial interventions include meaning-centered psychotherapy and dignity therapy (DT). Both have an underlying theme of attempting to reverse the narrative foreclosure for patients with serious illnesses and maintain a sense of meaning in life. In addition, patients can be referred to palliative care to enhance coping and decrease depressive symptoms. Dr. Harvey Chochinov has outlined a framework that clinicians can use to care for their patients in a compassionate manner to specifically combat meaninglessness. In DT, a generativity document is created for the patient and their loved ones as part of the treatment along with the opportunity to answer the dignity conserving question. Success of this route of intervention includes greater will to live, reductions in stress, and benefits perceived by family. This article aims to give a framework to treat patients with serious illnesses experiencing psychosocial and/or existential distress.

## Sample Patient Case

Mr. N, a 40-year-old gentleman, diagnosed with pancreas cancer underwent curative intent treatment. However, his cancer progressed, and he was not a surgical candidate. His prognosis is measured in months. He has a long-standing relationship with his primary care clinician (PCP) and has a visit to discuss his symptoms. He reports a decrease in appetite and significant weight loss and worries about his prognosis. He reports not being afraid to die but is struggling with leaving his wife and three young children. He worries how his wife will manage and is concerned that his two youngest children will not remember him. He has trouble sleeping and wonders why this is happening. He is angry and feels his diagnosis is unfair, questioning his faith.

As his PCP, you recognize that he is having existential distress related to his cancer and limited prognosis. How would you care for this patient and what resources are available? Mr. N is also trialing care from psychologists and is being followed by the outpatient palliative care team. Interventions such as asking the dignity conserving question (DCQ) and referring to providers trained in dignity therapy (DT) or meaning-centered psychotherapy (MCP) can be considered. Here is an example of a line of inquiry about his care goals using the DT questions and DCQ:

Physician: Mr. N, what do I need to know about you to provide the best care that I can?

Mr. N: I am 40 years old and have been married to my wife for 14 years. We have three children. I am worried about my two younger children forgetting about me.

Physician: Thanks for sharing that, I wonder when did you feel most alive?

Mr. N: I felt most alive when my first child (daughter-MJ) was born.

Physician: Tell me about your first child.

Mr. N: I have always wanted to be a father and to be the best father I could be to my children. When my daughter, my first child, was born, I was so happy that I could impact another human's life so much, educate them, love them, and be there for them in their happiest and saddest moments.

Physician: How did you accomplish these goals of impacting your children's life?

Mr. N: I worked as much as I could to provide for my family until the pain got too much. I always tried to be there for their soccer games, recitals, graduations, and other milestones. I was the one who taught my children how to tie their shoes.

After learning that Mr. N is feeling hopeless and that his life is meaningless, you decide to refer him to a provider trained in DT. Through an exploration of values, roles, and relationships, we eventually learned that Mr. N wanted to be everything he could be for his family. He felt demoralized by his disease, worried that he would not be able to care for his family in the way he valued. The team was able to identify what provided meaning and dignity to Mr. N and develop a plan to address his existential distress.

In creating this relationship, the clinician referred Mr. N to the specialty palliative care team that was able to complete a DT intervention. Mr. N's DT interview was transcribed, edited, and generativity document (GD) was given to Mr. N. Mr. N will share the document with his family and feels it will give his two younger children an opportunity to remember him.

## Introduction

When facing a serious illness, quality of life including physical, social, emotional, and spiritual well-being can be negatively affected.^1^ Psychosocial and existential distress, total pain (articulated as the sum of physical, spiritual, psychological, and social suffering^2^), suffering,^3^ grief,^4^ and demoralization^5,6^ are common in these patients. Care for a patient at this stage of illness centers on more than the physical symptoms.^7^

Suffering is personal and occurs with a threat to personal integrity. Existential pain can stem from lack of meaning.^3^ Caring for existential distress can be ambiguous, but nonetheless paramount to care for patients. Cancer patients, such as Mr. N, with limited life expectancy reported that dignity-related existential distress was a problem/major problem for 18.8% of patients.^8^

Existential and spiritual suffering are poorly understood by clinicians. Robust evidence is lacking in this population because this type of distress is challenging to study and these patients can be difficult to engage in research. Patients often find existential questions terrifying and anxiety provoking and avoid asking them.^9^ Surveys find a lack of knowledge and confidence in clinicians delivering primary palliative care.^10^ Even when specialty palliative care is provided, clinicians face their own moral distress and inadequately address this distress.^11^

## Discussion

A central question that some PCPs have incorporated into caring for patients with serious illnesses is the DCQ, which represents the essence of action elements of dignity conserving care (DCC): “What do I need to know about you as a person to take the best care of you that I can?”^12^ Patients value this question, and their answer is summarized in the medical record serving as a guide for clinicians.^12^ It is important to address this distress in primary care to maintain that important relationship and because palliative care is not widely available. Still, referral to specialty palliative care has been shown to improve quality of life and symptom management.^13^

Palliative care is specialized medical care for patients living with serious illness. Care is provided by an interdisciplinary team that focuses on the many aspects of serious illness. Evidence supports the role of palliative care supporting patients with advanced cancer, with studies showing an improvement in symptoms and in survival.^14^ Strengthening of patient coping efforts and strategies such as mindfulness and gratitude focused interventions, spiritual including pastoral care, and existential such as life review is a primary component of palliative care interventions. Patients seen by palliative care had more positive coping and less depressive symptoms.^15^

Importantly, palliative care is interdisciplinary team-based care including physicians, physician assistants, nursing, social work, chaplaincy, and music/integrative therapies. Delivering DCC with an interdisciplinary team allows an approach to whole-person caring and addressing total pain, alleviating suffering, and fostering healing.^16^

Serious illness can impact the sense of self, independence, and control leading patients to question the value or purpose of their lives, especially when they have lost the ability to fulfill roles that define them. Research literature has identified a patient's sense of meaning and purpose in life as one of most important influences on quality of life and psychological distress in patients with advanced cancer.^17^ Patients may benefit from more formal interventions such as DT or MCP, both forms of DCC.

DCC encompasses essential elements for clinicians to prepare and reflect on their practice. Harvey Chochinov, MD, outlines the clinician's attitude, behavior, compassion, and dialogue impact the experience of the patient, family, and clinicians.^18^ The characteristic attitude of DCC includes humility and curiosity to understand the patient's lived experience. The action of behavior is to be present and open to the lived experience. Compassion in DCC recognizes the vulnerability between patient and clinician. Lastly, the essential element of dialogue is the action of listening; listen to understand and to respond.

DT is a brief individualized psychotherapy that aims to relieve psychoemotional and existential distress and improve the experiences of patients whose lives are impacted by serious illness. DT offers patients an opportunity to reflect on their life with a trained facilitator and share their thoughts and memories with family. DT includes a detailed training manual and training course for providers. DT interviews entail two to three visits with a therapy provider.^19^ Participants are provided with a list of interview questions ahead of time (Table 1), to enable reflection on topics they most wish to discuss during the formal interview.

**Table 1.** Dignity Psychotherapy Question Protocol^19^

Tell me a little about your life history, particularly the parts that you either remember most or think are most important. When did you feel most alive?
Are there specific things that you would want your family to know about you, and are there particular things you would want them to remember?
What are the most important roles you have played in life (family roles, vocational roles, community-service roles, etc.)? Why were they so important to you, and what do you think you accomplished in these roles?
What are your most important accomplishments, and what do you feel most proud of?
Are there particular things that you feel still need to be said to your loved ones, or things that you would want to take time to say once again?
What are your hopes and dreams for your loved ones?
What have you learned about your life that you would want to pass along to others? What advice or words of guidance would you wish to pass along to your (son, daughter, husband, wife, parents, others)?
Are there words or perhaps even instructions you would like to offer your family to help prepare them for the future?
In creating this permanent record, are there other things that you would like included?

The interview is transcribed, and the transcription is edited for clarity and flow to produce a GD. This document is then shared with the participant for validation and further edited as needed to ensure all intended content is included. The final version of this document is then provided to the participant to share with whomever he/she chooses.

DT participants have reported high rates of intervention satisfaction, improved sense of dignity, increased sense of purpose and meaning, greater will to live, decreased sense of suffering, and perceived benefits to the family.^20^ There have been five randomized studies of DT with varying results.^21^ Two of these trials included patients with high baseline psychological distress, with one showing a statistically significant decrease on patients' anxiety and depression measures from baseline.^22^ The other showed statistically significant decreases in anxiety but not in depression pre–post DT.^23^ Overall, nonrandomized studies reported statistically significant improvements on existential and psychosocial measurements, such as suffering and depression.^20^

MCP was developed to address the loss of spiritual well-being or sense of meaning in life and the existential distress that arises in patients with advanced cancer. MCP has been studied both in group and in individualized settings. One study of meaning-centered group therapy noted improved spiritual well-being and quality of life and significantly greater reductions in depression, hopelessness, desire for hastened death, and physical symptom distress compared with those receiving supportive group psychotherapy.^24^

Individual MCP demonstrated greater improvements in spiritual well-being and improved symptom burden and symptom-related distress. However, these improvements were short lived and no longer statistically significant at two months.^25^ This intervention is provided in 7–8 weekly sessions, although there has been research into an abbreviated three-session approach for palliative care patients.^26^ Availability remains a barrier to providing DT and MCP.

PCPs develop long standing relationships with patients, potentially addressing the physical and/or emotional/existential distress related to their illness. Although these conversations can be challenging, the DCC model and DCQ can help guide the conversation and identify the patient's values and goals. Although MCP was considered as well, sustained improvements in existential distress were prioritized. The art of listening to their story and remaining present provide a space to restore meaning and purpose, find hope that can lead to decreased suffering.

PCPs can also refer similar patients to a specialty palliative care team trained in DT or MCP. Our experience with Mr. N, the 40-year-old man with late-stage pancreas cancer, is illustrative. Mr. N feels helpless and fears that he will be forgotten by loved ones. Caring for a patient facing existential distress is a challenge, and Chochinov's DT and DCC may be therapeutic for Mr. N. The DCC and DT focus on knowing who the patient is, what roles he plays, what he is the proudest of, and his life story in the context of approaching the end of life.

## Abbreviations Used

DCC: dignity conserving care
DCQ: dignity conserving question
DT: dignity therapy
GD: generativity document
MCP: meaning-centered psychotherapy
PCP: primary care clinician
